# Cytotoxicity of Endodontic Irrigants on Human Periodontal Ligament Cells

**DOI:** 10.22037/iej.v13i3.20438

**Published:** 2018

**Authors:** Hamed Karkehabadi, Hosnieh Yousefifakhr, Saeede Zadsirjan

**Affiliations:** a *Department of Endodontics, Dental School, Hamadan, Iran; *; b *Department of Periodontics, Faculty of Dentistry, Borujerd Branch, Islamic Azad University, Borujerd, Iran; *; c *Department of Endodontics, Dental School, Shahid Beheshti University of Medical Sciences, Tehran, Iran*

**Keywords:** Cytotoxicity Test, MTT Tetrazolium, Periodontal Ligament, Root Canal Irrigants

## Abstract

**Introduction:**

Root canal irrigation has an extremely important role in the success of endodontic treatment. During endodontic treatment, the irrigants will be in contact with pulpal and periapical tissues. The purpose of this study was to clarify the potential toxicological implications of NaOCl, EDTA, MTAD, CHX and QMix on periapical and periodontal tissues.

**Methods and Materials:**

Cytotoxicity of solutions was evaluated on cultured human periodontal ligament (hPDL) that were carefully removed from the middle third of premolar roots. Cytotoxicity of the materials was assessed after 1, 5 and 15 min of exposure using the Mosmann’s Tetrazolium Toxicity (MTT) assay. Optical density of the solution was read at 540-690 nm wavelength. The intensity of color generated correlated with the percentage of viable cells. Data were statistically analyzed using repeated measures ANOVA followed by Bonferroni test.

**Results:**

The mean percentage of viable cells in all experimental groups was significantly different from sterile saline groups at all time points (*P*<0.0001). The mean percentage of viable cells significantly decreased over time in MTAD and NaOCl groups. The lowest and highest cytotoxicity belonged to MTAD and EDTA groups, respectively at all the time points (*P*<0.05).

**Conclusion:**

MTAD had the lowest cytotoxicity compared to NaOCl, CHX, QMix and EDTA. These impacts have been time dependent. These irrigation fluids may cause unfavorable effects on vital tissues.

## Introduction

The fulfillment of endodontic treatment depends at the eradication of microbes from the root canal system and the following prevention of reinfection. Root canal irrigation has an extremely important role in the success of endodontic treatment [1]. A really perfect root canal irrigant should be nontoxic, with a broad antimicrobial spectrum and the capacity to dissolve necrotic pulp tissue, inactivating endotoxins, and either prevent the formation of a smear layer or dissolve it [2-5]

An extensive variety of irrigating solutions are available for endodontic use, such as NaOCl, EDTA and chlorhexidine (CHX) [6]. NaOCl has been widely prescribed as irrigation solution to aid in the chemomechanical debridement of the root canal system due to its dissolving activity on pulp tissue and its antimicrobial properties. Because of its substantive antimicrobial properties, CHX has become an effective oral antimicrobial agent for use in periodontal treatment and caries prevention and a remedial agent for other oral infections [7, 8]. Ethylenediaminetetraacetic acid (EDTA) is effective for removing the inorganic component of the smear layer [9, 10]. 

Beside many commonly used irrigating solutions such as NaOCl and CHX, there are many commercial multifunctional mixtures accessible for this purpose. QMix (Dentsply Tulsa Dental Specialties, Tulsa, OK, USA) is a 2-in-1 solution containing a bisguanide antimicrobial agent (2% CHX) and a polyaminocarboxylic acid calcium-chelating agent (17% EDTA) [11]. MTAD (Dentsply, Tulsa, OK, USA) is a mixture of a tetracycline isomer, citric acid and a detergent (tween 80). This solution have been effectively used in disinfection of root canal system [12].

During endodontic treatment, the irrigating solution will be in contact with pulpal and periapical tissues. Debris as well as irrigating solutions may also be pushed beyond the apical foramen and cause further periapical complications [13]. Bajrami *et al.* [14] evaluated *in vitro* cytotoxic damage induced by NaOCl, CHX and MTAD at different dilution on periodontal ligament fibroblast cells. They demonstrated that MTAD showed similar cytotoxicity to 3% NaOCl at all-time points at both dilutions and indicated that 2% CHX was more cytotoxic than the other 2 irrigants. According to Zhang *et al*. [15] study, there was a correlation between NaOCl concentration and its cytotoxicity, too. Yasuda *et al*. [16] reported that MTAD had minimal cytotoxicity against MC3T3 and periodontal ligament cells compared to conventional irrigants.

The purpose of this study was to clarify the potential toxicological implications of NaOCl, EDTA, MTAD, CHX and QMix on periapical and periodontal tissues. Since a definitive objective in endodontic treatment is the recovery of periapical tissues, the goal was to assess the cytotoxicity of different irrigation solutions on cultured human periodontal ligament (PDL) cells because these cells are responsible for normal maintenance and the regeneration of the periodontium [13]. 

## Materials and Methods

Cytotoxicity of solutions was evaluated on cultured hPDL, fibroblast cells in research laboratory, Hamedan University, faculty of dentistry, Hamedan, Iran. 

The study protocol was approved in the ethics committee of Hamedan University of Medical Sciences (ID: IR.UMSHA.REC.1396.496). Human PDL cells [17, 18] were cultured from the roots of premolar extracted for orthodontic treatment. To avoid contamination from the gingiva, periodontal ligament was carefully removed from the middle third of root. The fragments were grown in 96-well plates containing Dulbecco’s modified Eagle’s medium (DMEM), supplemented with 10% fetal bovine serum (FBS) (Gibco, Grand Island, NY, USA) and antibiotics. Culture were incubated at 37^◦^C in a humidified atmosphere, 95% air and 5% Co_2_ for 24 h in water based incubator [8].

To obtain more cells, cells were re-cultured in culture medium containing 15% FBS. This cell line was cultured in culture medium containing 10% DMEM/bovine serum in sterile cell culture flasks (SPL Life, Science, Gyeonggi-do, South Korea). During the process of cell culture, the culture medium was refreshed every 2-3 days and cells were passaged after one week. After four passages, cells reached adequate confluence for cytotoxicity testing. Next, stem cells were transferred to 24-well plates and randomly divided into 6 experimental groups and subjected to BioPure MTAD (Dentsply, Tulsa Dental, Tulsa, OK, USA), QMixTM 2 in 1 (Dentsply, Tulsa Dental, Tulsa, OK, USA), 17% EDTA (MD-cleanser, Meta Biomed, Chungju, Korea), 2% CHX (Clorhexidina S, Dentscare LTDA, Joinville, Sc, Brasil), 5.25% NaOCl (Sehat, Tehran, Iran) and sterile saline. Stem cells cultured in DMEM were used as a control group. Cytotoxicity of the materials was assessed after 1, 5 and 15 min of exposure using the Mosmann’s Tetrazolium Toxicity (MTT) assay.

The MTT solution was prepared by dissolving 5 mg of 3- (4,5-dimethyl-2-thiazolyl)-2, 5-diphenyl-2H-tetrazolium bromide (Sigma-Aldrich Co., St. Louis, MO, USA) in 1 mL of PBS. After filtering, this solution was diluted 1 to 10 using DMEM; 400 L of the diluted MTT solution was added to each well and plates were incubated at 37 C under 5% CO_2_ and 95% humidity for 4 h. After dissolution of formazan crystals, optical density of the solution was read at 540-690 nm wavelength using an Elisa Reader (Bio Tek, Winooski, VT, USA). The intensity of color generated correlated with the percentage of viable cells. Data were analyzed at different time points *via *repeated measures ANOVA followed by Bonferroni test. Level of significance was set at 0.05. 

**Table 1:** The percentage of viable cells in the experimental groups during 1, 5 and 10 min

**Experimental Groups**	**1 min**	**5 min**	**10 min**	***P*** **-value** *
**MTAD**	42.52 (10.06) Aa	34.57 (11.1) Ab	31.91 (10.68) Ab	0.031
**EDTA**	16.34 (2.28) C	15.60 (3.19) C	14.97 (3.81) C	0.758
**Chlorhexidine**	28.21 (4.06) B	24.38 (4.2) B	24.21 (4.51) B	0.218
**QMIX**	21.37 (3.83) D	19.42 (3.61) C	18.42 (3.23 D	0.371
**Hypochlorite**	37.84 (6.12) Aa	24.54 (3.75 Bb	22.83 (2.86) Bb	0.000
**Normal Saline**	86.63 (13.69)	104.10 (19.95)	116.65 (23.91)	0.05
***P*** **-Value** **	0.000	0.000	0.000	

*. *P*-value (Repeated measures test) comparison of the effect of irrigants over time;

**. *P*-value (One way ANOVA) comparison of viability of irrigants;

a , . (Bonferroni) the effect of viability of irrigants over time;

A , , , . (Bonferroni) the effect of viability between irrigants

## Results

The Bonferroni test was used to compare the viability of different groups. The results showed that the difference in the mean percentage of viable cells between the study groups and the control at 1, 5 and 10 min was statistically significant (*P*<0.0001). MTAD showed the highest cell viability (*P*<0.05) but EDTA significantly had the lowest cell viability compared to other irrigants (*P*<0.05) at 1, 5 and 10 min. 

Over time, no statistically significant change occurred in the mean number of viable cells in the EDTA, CHX and Qmix but in MTAD and NaOCl samples the mean number of viable cells decreased. The difference in the percentage of viable cells between 1 and 15 min time points was only significant in NaOCl and MTAD (*P*<0.05). The highest and the lowest cytotoxicity belonged to EDTA and sterile saline group, respectively.

The mean number of cell viability comparison in different groups at 1, 5 and 15 min are shown in Table 1.

## Discussion

This *in vitro* study was conducted to assess the cytotoxicity of NaOCl, EDTA, MTAD, CHX and QMix on human periodontal ligament cells using MTT assay. Complete debridement of the root canal system with the use of proper irrigants, eliminate the quantity of microorganisms and increases chances for successful root canal therapy [15]. The toxicity of materials used in endodontic therapy are precise concern because damage or irritation could cause degeneration of the periapical tissue and delayed wound healing. Ideal endodontic irrigating solution should be selectively toxic and act as an antimicrobial agent but with low periradicular tissue toxicity. MTT is well set up for cytotoxicity analysis of materials, being used initially for cell viability analysis in the 1980s [14]. This method assesses the ability of viable cell in changing the water-soluble tetrazolium salts to the insoluble formazan crystals *via* the activity of mitochondrial dehydrogenase enzymes. MTT assesses the cytotoxicity of dental materials based on the changes in the number of viable cells, cell metabolism and cell morphology. In this method, cell damage is underestimated and only cell death, in the apoptotic phase, is detected when cellular metabolism significantly decreases [14-16, 19]. According to our results, MTAD, QMix, NaOCl, EDTA and CHX solutions all induced cytotoxicity in human periodontal ligament cells and these impacts were time-dependent. The rate of cytotoxicity the tested irrigants in ascending order was EDTA, QMix, CHX, NaOCl and MTAD. Evaluation of cytotoxicity of material *in vitro* is completely cellular. Cell culture was compared to the periapical tissue which are highly susceptible to the toxic effect of materials [14]. *In vitro* tests characterized by quickness, inexpensiveness, sensitivity and reproducibility, can be performed either directly or through analysis [20]. Unfortunately, the results acquired by this type of assessment are not adequate for a conclusive clinical evaluation because under *in vivo* conditions, materials are diluted with body fluids and their concentration decrease [21]. Also, they are reduced by the function of phagocytes, vascular and lymphatic systems, the inhibitory effect of dentin on irrigants must be taken [22, 23]. Thus, in equal concentrations, the cytotoxicity of materials reduces over time in the clinical setting compared *in vitro* [14, 24]. 

The study also demonstrated that MTAD was less cytotoxic than the other tested irrigants. This finding is in accordance with the results separately reported by Yasuda *et al.* [16], Zhang *et al.* [15] and Ring *et al.* [25] that stated higher biocompatibility of MTAD in comparison with NaOCl 5.25% and EDTA 17%. 

The observations from the study confirmed that the highest cytotoxicity belonged to EDTA, followed by QMix, CHX and NaOCl. The present results are in contrast with previous studies [26-28] which indicated that NaOCl is more cytotoxic than EDTA but Serper *et al.* [29] found that the cytotoxicity of EDTA was remarkable at any dilution as evaluated by MTT assay. These findings are consistent with Koulaouzidou *et al*. [30] who reported that at 17%, 15% and 1%, EDTA demonstrated severe cytotoxity under *in vitro* conditions. Amaral *et al.* [31] showed that EDTA probably exerted a direct effect on macrophages, promoting alterations on their cell membranes caused by chelator ions, such as Ca^2+^ and Mg^2+^, and accelerating the apoptotic process as these divalent cations are considered cofactors to several enzymatic reactions. These findings are in agreement with Segura *et al.* [32], who reported an inhibitory effect on vasoactive intestinal peptides (VIP) caused by EDTA. They concluded that EDTA reduced the VIP binding to macrophage membranes that are responsible for the modulation of periapical immune response. 

CHX is a toxic agent that binds to cell plasma membrane and increases its permeability, permitting the leakage of lysosomal enzymes [33]. *In vitro* studies about cytotoxicity recommended that CHX had a higher toxicity in cell cultures than NaOCl [22]. Nevertheless, they are in contrast with a study [15] which found that CHX, EDTA and NaOCl display comparable cytotoxicity andwith other studies by Yasuda *et al.* [16] and Mollashahi *et al*. [34] who stated that CHX is less cytotoxic than NaOCl and EDTA. 

Alkahtani *et al*. [35] compared the cytotoxicity of NaOCl and QMix, which contains both EDTA and CHX, they determined that both solutions were toxic to human bone marrow MSC, but in a different mode. EDTA, which is the second QMix component, is also known to be cytotoxic, perhaps due to its chelating impact and the accentuated drop in pH that it causes [9]. However, *in vivo* investigations reported that CHX or QMix are less toxic than NaOCl [33, 36].

The less cytotoxicity of tested irrigants in this study related to NaOCl and MTAD. The antimicrobial effectiveness and cytotoxicity of sodium hypochlorite are based on its high pH [37]. According to Saghiri *et al*. [38] the pH of NaOCl added to the medium approached the neutral pH values in less time than the other irrigants which may be due to NaOCl dispersal ability in aqueous medium. Lowering the pH of the root canal irrigant (*e.g*. sodium hypochlorite) has some advantages such as increased efficacy, lower toxicity to vital host tissues, and increased antibacterial ability [39].

However, the result of these studies cannot be compared with the results of the present study due to different methodology and concentration of solution used. The cytotoxicity of irrigants relied on the exposure dose, composition of the exposure medium and length of exposure [40]. Estimation of cytotoxicity is absolutely cellular at this *in vitro* study, so our outcomes cannot be directly generalized to *in vivo* studies. New investigations on root canal irrigants should be done in animals and then in humans to assess their cytotoxicity and *in vivo* biocompatibility.

## Conclusion

MTAD had the lowest cytotoxicity compared to NaOCl, CHX, QMix and EDTA. These impacts have been time dependent. These irrigation fluids may cause unfavorable effects on vital tissues. Its clinical significance needs to be evaluated further because exposure time, exposure surface area and concentration are vital factors affecting the toxicity effect.
